# Trends in Hospital Admissions for Patients with Amyotrophic Lateral Sclerosis: Insights from a Retrospective Cohort Study in a Province in Northern Italy

**DOI:** 10.3390/life14080941

**Published:** 2024-07-27

**Authors:** Giulia Gianferrari, Elisabetta Zucchi, Ilaria Martinelli, Cecilia Simonini, Nicola Fini, Salvatore Ferro, Andrea Mercati, Laura Ferri, Tommaso Filippini, Marco Vinceti, Jessica Mandrioli

**Affiliations:** 1 Neurosciences PhD Program, Department of Biomedical, Metabolic and Neural Sciences, University of Modena and Reggio Emilia, 41126 Modena, Italy; 177540@studenti.unimore.it (G.G.); elisabetta.zucchi@unimore.it (E.Z.); lauraferri091@gmail.com (L.F.); 2 Department of Neurosciences, Azienda Ospedaliero Universitaria di Modena, 41126 Modena, Italy; martinelli.ilaria@aou.mo.it (I.M.); ceciliasimonini24@gmail.com (C.S.); fini.nicola@aou.mo.it (N.F.); 3 Clinical and Experimental Medicine PhD Program, Department of Biomedical, Metabolic and Neural Sciences, University of Modena and Reggio Emilia, 41125 Modena, Italy; 4 Department of Hospital Services, Emilia Romagna Regional Health Authority, 40127 Bologna, Italy; salvatore.ferro@regione.emilia-romagna.it; 5 Specific Training Course in General Medicine, University of Siena, 53100 Siena, Italy; andrea.mercati@tim.it; 6 Department of Biomedical, Metabolic and Neural Sciences, University of Modena and Reggio Emilia, 41125 Modena, Italymarco.vinceti@unimore.it (M.V.)

**Keywords:** amyotrophic lateral sclerosis, hospitalization, procedures, ventilation, nutritional support, emergency

## Abstract

ALS is characterized by a highly heterogeneous course, ranging from slow and uncomplicated to rapid progression with severe extra-motor manifestations. This study investigated ALS-related hospitalizations and their connection to clinical aspects, comorbidities, and prognosis. We performed a retrospective cohort study including patients residing in Modena, Italy, newly diagnosed between 2007 and 2017 and followed up until 31 December 2022. Data were obtained from the Emilia Romagna ALS registry, regional hospitals, and medical records. Among the 249 patients, there were 492 hospital admissions, excluding those for diagnostic purposes; 63% of the patients had at least one hospitalization post-diagnosis, with an average stay of 19.90 ± 23.68 days. Younger patients were more likely to be hospitalized multiple times and experienced longer stays (44.23 ± 51.71 days if <65 years; 26.46 ± 36.02 days if older, *p* < 0.001). Patients who were hospitalized at least once more frequently underwent gastrostomy (64.97%) or non-invasive (66.24%) and invasive (46.50%) ventilation compared to those never hospitalized (21.74%, 31.52%, 13.04%, respectively, *p* < 0.001 for all). Emergency procedures led to longer hospitalizations (62.84 ± 48.91 days for non-invasive ventilation in emergencies vs. 39.88 ± 46.46 days electively, *p* = 0.012). Tracheostomy-free survival was not affected by hospitalizations. In conclusion, younger ALS patients undergo frequent and prolonged hospitalizations, especially after emergency interventions, although these do not correlate with reduced survival.

## 1. Introduction

Amyotrophic lateral sclerosis (ALS) is a progressive neurodegenerative disease that impacts motor neurons in the brain and spinal cord. This debilitating disease is characterized by progressive muscle paralysis, resulting in accumulating disability and death. ALS is typically marked by an average survival of 3–5 years. However, the disease exhibits a highly heterogeneous natural history, with variations in disease progression, survival rates, and complications [1]. Hospitalizations are common among ALS patients and represent the third largest contributor to the cost of care, following home care and both non-invasive (NIV) and invasive ventilation (IV) [2]. Hospitalizations frequently involve managing respiratory complications or infections, as well as performing procedures related to nutrition and ventilation [2,3,4]. Those hospitalized for respiratory failure face a higher in-hospital mortality rate compared to the general population [2,3]. Complications such as deteriorating general condition, malnutrition, and respiratory insufficiency increase the risk of severe infections, including sepsis [5,6]. While some medical interventions related to the disease necessitate hospitalization, studies indicate that certain complications, like respiratory infections, can be effectively managed at home with proper care and support, including NIV, manual and/or mechanical cough assistance, and monitoring of arterial oxygen saturation [7]. Prolonged hospitalization due to preventable complications should be avoided, considering the potential burdens on patients and their families in terms of quality of life and possible complications that can exacerbate an already fragile clinical picture [5]. Despite the significant consequences of hospitalization in patients with ALS, this issue has been relatively underexplored in the literature. In this study, we evaluate the frequency and main causes of hospitalization in ALS patients, investigating their relationship with clinical aspects, comorbidities, and prognosis. The aim is to identify potential prognostic or favorable factors for hospitalization to improve the organization and care of patients and their families.

## 2. Materials and Methods

### 2.1. Study Population and Data Collection

We conducted a retrospective cohort study on patients with ALS in the province of Modena, diagnosed from 2007 to 2017. The follow-up period was for at least 5 years, with the last observation date set at 31 December 2022. The patient’s profiles included variables such as sex, years of school education, body mass index (BMI) at diagnosis, age at onset and diagnosis, diagnostic delay, site of onset, El Escorial criteria classification, clinical phenotype (bulbar, classical, flail arm/flail leg, upper motor neuron predominant (UMNp), respiratory ALS), presence of dementia, disease duration, date and cause of death, and treatment with riluzole. Additionally, we assessed the ALS Functional Rating Scale—Revised (ALSFRS-R) at diagnosis, progression rate (assessed as the monthly decline in ALSFRS-R total score from onset to diagnosis), and respiratory function measured by forced vital capacity (FVC) [8]. Details of hospitalizations, including causes, events, complications, durations, and outcomes, were recorded from medical records. Pharmacological treatments administered during hospital admission were not recorded. The province of Modena hosts seven hospitals and one ALS Center located at Azienda Ospedaliero-Universitaria di Modena. As of 31 December 2022, the population of the province of Modena was 706,892 (https://statistica.regione.emilia-romagna.it/servizi-online/statistica-self-service/popolazione/popolazione-per-eta-e-sesso, accessed on 1 March 2024) [9]. Nutritional (percutaneous endoscopic gastrostomy—PEG) and respiratory (non-invasive and invasive ventilation—NIV and IV) procedures and the timing of their execution with respect to symptom onset were also recorded [10]. Comorbidities at diagnosis were categorized as previously reported [11]. The data were integrated from the population-based registry of ALS patients resident in Emilia Romagna Region [12], cases discharged with the code 335.2 of the International Classification of Diseases (ICD), 9th revision, or the corresponding G12.21 code of the ICD, 10th revision, from regional hospitals, and medical records.

### 2.2. Statistical Analyses

For statistical analysis, continuous variables were presented as means with standard deviations (SD), while categorical variables were reported as absolute numbers and relative frequencies (percentages). Group comparisons were conducted using two-tailed t-tests and ANOVA for continuous variables, applicable, respectively, for two groups or multiple groups. Chi-square tests were employed for comparisons between categorical variables. Pearson’s test was used for correlation analyses. Poisson regression analysis was conducted to evaluate the influence of clinical features and intervention on the number and length of hospitalizations. Survival analysis was conducted using Kaplan–Meier curves, and the log-rank test was applied for univariate analyses, while multivariate analyses were performed using the Cox regression model (stepwise backward method). A *p*-value of less than 0.05 was considered statistically significant. Data analysis was carried out using the STATA statistical package version 15 (StataCorp. 2017, StataCorp LLC., College Station, TX, USA).

## 3. Results

### 3.1. Patient’s Clinical Features

Two hundred forty-nine patients were included in the study. Their clinical and demographic features are shown in Table 1.

**Figure 1:**
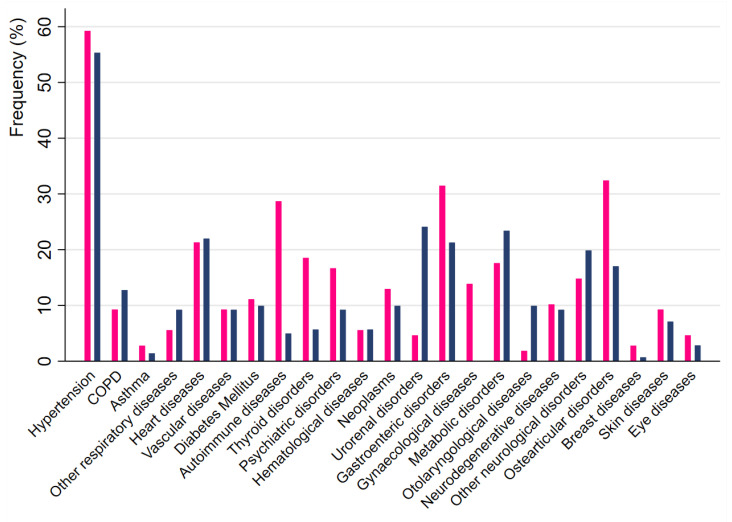
Frequency of comorbidities in the study cohort of ALS patients according to sex (pink bars = females; blue bars = males).

Women presented with lower ALSFRS-R total scores at diagnosis than men; the difference remained after adjusting for education, diagnostic delay, and age at onset (95%CI 0.52 to 4.12, *p* = 0.012). Among the comorbidities at diagnosis, autoimmune or immune-mediated diseases were more frequent in females than males (28.70% vs. 4.96%, *p* < 0.001), as were thyroid and osteoarticular diseases (18.52% vs. 5.67%, *p* = 0.001 and 32.41% vs. 17.02%, *p* = 0.005, respectively). Conversely, urorenal and otorhinolaryngological diseases were more common in males (24.11% vs. 4.62%, *p* < 0.001, and 9.92% vs. 1.85%, *p* = 0.010, respectively) (Figure 1). For all other comorbidities, there were no significant sex differences.

The type or number of comorbidities did not affect the probability of hospitalization (Table 2).

### 3.2. Hospitalization: Frequency, Duration, Causes, and Events Related to Hospital Admission in Patients with ALS

In total, 492 hospital admissions were observed in the province of Modena. The majority of these hospitalizations were concentrated at the hospitals of Azienda Ospedaliero-Universitaria of Modena (n = 464, 94.31%), with only a few admissions to other smaller hospitals (n = 28, 5.69%). Excluding hospitalizations for diagnosis purposes, 157 patients (63.05%) had at least one additional hospital admission, with an average stay of 19.90 ± 23.68 days. Additionally, 89 (35.74%) had at least two hospitalizations, 52 (20.88%) had at least three, 36 (14.46%) had at least four, and 22 (8.83%) had five or more hospitalizations. The clinical–demographic features of patients who had never been hospitalized after diagnosis compared with those who had been hospitalized are shown in Table 2. While there were no significant differences in the number of hospitalizations between males and females, younger patients were more likely to be hospitalized than older ones. Furthermore, patients undergoing PEG, NIV, and IV were hospitalized more frequently than those who refused nutritional or respiratory support (Table 2). Poisson regression analysis confirmed that age at onset (Coef: −0.01, 95% CI: −0.02 to −0.01, *p* = 0.002), NIV (Coef: 0.21, 95% CI: 0.04 to 0.38, *p* = 0.014), and tracheostomy (Coef: 0.62, 95% CI: 0.43 to 0.80, *p* < 0.001) affected the number of hospitalizations. The use of PEG or BMI at diagnosis did not significantly affect the number of hospitalizations.

The average number of days spent in hospital per patient was 32.67 ± 42.91 days. Patients with at least two hospitalizations averaged 41.54 ± 30.86 days, those with three hospitalizations 55.82 ± 37.33 days, those with four hospitalizations 68.53 ± 36.79 days, and those hospitalized five or more times averaged 94.41 ± 45.29 days. The patients who underwent emergency supportive procedures spent more days in the hospital (Table 3).

Poisson regression analysis confirmed that age at onset (Coef: −0.01, 95% CI: −0.01 to −0.01, *p* < 0.001), NIV (Coef: 0.32, 95% CI: 0.27 to 0.38, *p* < 0.001), PEG (Coef: 0.27, 95% CI: 0.21 to 0.33, *p* < 0.001), and tracheostomy (Coef: 1.44, 95% CI: 1.38 to 1.50, *p* < 0.001), affected the global number of days of hospitalization.

Older patients were hospitalized for significantly shorter periods (r = −0.234, *p* = 0.0002), whereas the length of hospitalization did not correlate with a higher progression rate at diagnosis (r = 0.0275, *p* = 0.671).

**Figure 2:**
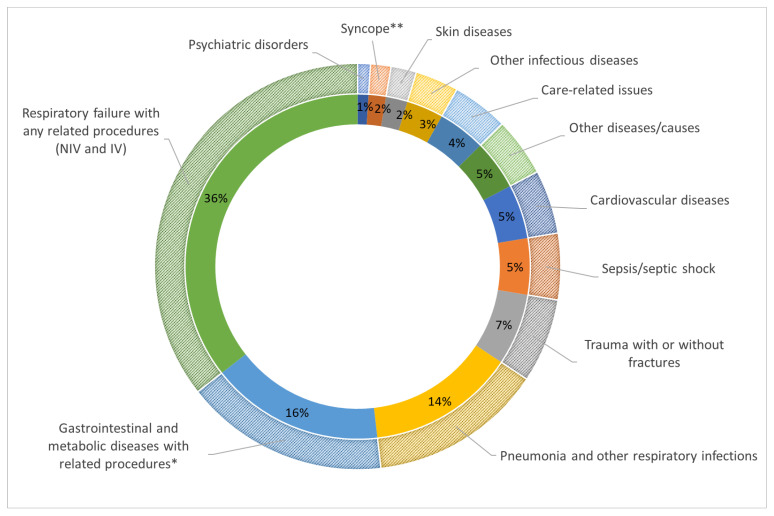
Causes of hospitalization among the patients of the study. NIV = non-invasive ventilation; IV = invasive ventilation. * Nutritional support (enteral or intravenous), hydration, electrolytes infusion. ** Loss of consciousness not due to respiratory or infectious diseases (e.g., vagal syncope).

The most common reason for hospital admission in our sample was respiratory failure, often accompanied by related procedures such as adaptation to NIV or tracheotomy for invasive ventilation. Respiratory failure and related interventions accounted for a total of 175 admissions (35.58%). Other common causes of hospital admission included gastrointestinal disturbances (including dysphagia and malnutrition) and metabolic disorders (n = 80, 16.26%), pneumonia and other respiratory infections (n = 68, 13.82%), trauma (n = 33, 6.71%), and sepsis (n = 26, 5.28%). Figure 2 shows the major causes of hospitalization in our patient cohort.

Additionally, we assessed events and procedures that, although they did not prompt the hospital admission, complicated or defined the hospital stay for each admission: the most frequent event during hospitalization was the placement or change of the tracheostomy cannula for IV, occurring in 56 admissions (21.05%). Other frequent events included PEG placement or metabolic decompensation in 31 hospitalizations (11.65%), sepsis or septic shock in 25 admissions (9.38%), and NIV placement in 23 hospitalizations (5.28%). Death occurred in 43 hospitalizations (16.17%).

### 3.3. Hospitalization, Prognosis, and Survival

Median tracheostomy-free survival from onset was 26.24 months (95% CI 23.35–30.67), and it did not differ significantly between subjects with more frequent or longer hospital stays. At 36, 48, and 60 months, 35.74%, 27.42%, and 21.4% of subjects, respectively, were alive.

Apart from already known disease features associated with tracheostomy-free survival, neither the number nor length of hospitalizations nor comorbidities had any influence on survival in our cohort. The results of the univariate analysis of factors influencing tracheostomy-free survival in our patient cohort are shown in Table 4.

Multivariate analysis showed that factors impacting on tracheostomy free survival were diagnostic delay (months; HR 0.95, 95% CI 0.93–0.97, *p* < 0.001), age at onset (years; HR 1.03, 95% CI 1.02–1.05, *p* < 0.001), FVC at diagnosis (%; HR 0.99, 95% CI 0.98–1.00, *p* = 0.005), phenotype (UMNp with respect to bulbar; HR 0.25, 95% CI 0.12–0.55, *p* < 0.001), and weight loss at diagnosis (kg, HR 1.03, 95% CI 1.01–1.06, *p* < 0.001).

## 4. Discussion

ALS exhibits a variable course in terms of survival, progression speed, and complications. This variability can partly be attributed to the type of care received: it is well-established that a multidisciplinary approach and the implementation of supportive procedures for ventilation and nutrition are linked to increased survival [13,14,15,16]. Most respiratory and nutritional support procedures require hospitalization, which can occur urgently following respiratory failure or infectious events, or electively [5].

In agreement with the literature [2], hospitalizations occurred quite frequently among the ALS patients of our cohort: 63% of them had at least one hospital admission, after diagnosis, with an average duration of nearly 20 days. The main difference between patients who had never been hospitalized and those who had revolved around age at diagnosis. Younger patients were more likely to be hospitalized compared to their older counterparts. Among the reasons for this difference, we may hypothesize, is a greater willingness to undergo medical procedures to support respiration and nutrition and possibly better overall health, which makes them suitable candidates for such interventions. Moreover, younger patients may have better access to healthcare resources and supportive care services, including augmentative and alternative communication, leading to more proactive management, including hospitalization when necessary. However, we found no significant differences between the two groups regarding the coexistence of frontotemporal dementia or other comorbidities (even after adjusting for age), which are therefore not a limiting factor for access to hospital care.

Forty-nine percent of the patients agreed to undergo PEG, an invasive support procedure that requires hospitalization for placement and periodic checks to ensure its proper functioning. While the use of nutritional support can facilitate symptom control, prevent complications such as aspiration pneumonia, and positively influence survival [15,17], this procedure is not without potential complications, morbidity, and mortality [18], as demonstrated by multiple and longer hospitalizations experienced by patients with PEG in our cohort.

In our cohort, 53% of the patients used NIV, and about 22% of them were never hospitalized. Indeed, the adaptation of NIV at home or in a day hospital setting is an available option and is practiced at our ALS Center. As reported in the literature, the initiation of NIV typically occurs in the hospital; however, attempts at adaptation in a day hospital or home setting have been associated with good treatment adherence and effective correction of nocturnal hypoxemia [19,20]. These data should encourage an improvement in home management and telemonitoring for patients with chronic respiratory failure [20,21].

As far as IV is concerned, 34% of the patients underwent tracheostomy: this percentage is higher than reported in the general literature [22,23,24] but similar to what is reported in the Japanese population [25] and may relate to the high level of social assistance provided in the Emilia Romagna Region and to cultural acceptability [26]. Individuals opting for invasive mechanical ventilation are typically younger, often have young children, and exhibit higher levels of education and socioeconomic status. Moreover, these patients consistently display notable optimism and maintain positive perspectives on their daily lives [23,25]. As expected, patients undergoing ventilation procedures experienced the highest number and duration of hospitalizations. Consistently, we observed a greater number and longer duration of hospitalizations in younger individuals, who are more likely to accept the use of invasive ventilatory support [25]. Despite the positive effects of ventilatory support procedures on survival [14], the literature suggests that patients more frequently undergo hospitalization and die due to infectious complications [27,28].

The most frequent cause of hospitalizations was respiratory failure, often managed with NIV or IV, followed by pneumonia, as commonly reported in the literature [2]. The management of respiratory insufficiency and infectious disorders, contrary to recommendations, often necessitates emergency hospitalizations [2]. In our sample, while most NIV initiations were elective (>70%), the use of invasive ventilation primarily occurred during emergencies, which correlated with increased complications [5] and longer hospitalizations [29]. In agreement with the literature, while emergency admissions primarily address acute exacerbations of ALS symptoms, elective admissions focus on proactive management of the disease through supportive care, such as NIV or PEG [30].

As previously reported, palliative care and advanced end-of-life directives have been available options in our region for many years [26]. However, based on our experience, many patients change their minds as they approach the final stages of their illness, and some remain uncertain about invasive procedures until an acute event occurs. Despite that, withdrawals of IV are very rare, even following the enactment of Italian Law 219/2017. This legislation, a first in Italian law, guarantees patients the right to request the withdrawal of life-sustaining treatments, including mechanical ventilation [31].

While malnutrition, respiratory failure, mechanical ventilation, and invasive devices may all increase the risk of infection and sepsis [6], the quantity and nature of comorbidities did not influence the frequency of hospitalizations. Accordingly, data on hospitalization causes suggest that more frequent hospitalizations can be attributed to disease progression rather than other comorbidities.

The median survival from onset to death or tracheostomy was approximately 26 months, aligning with the general ALS population [32] when observed over an extended period. Our previous studies on the same population demonstrated longer tracheostomy-free survival during shorter observation periods, where a smaller proportion of patients died (65% versus 90% in the current study) [26,33]. This emphasizes the necessity of sufficiently (at least 2 years) long observation periods to produce reliable estimates of patient survival.

In multivariate analysis, independent prognostic factors for tracheostomy-free survival, as previously described, include onset age, with the risk of death increasing with age [11,34,35]. The disease phenotype also influences survival; flail leg and UMN-p phenotypes are associated with longer survival, while the bulbar phenotype is linked to reduced survival due to earlier impairment of breathing and swallowing muscles [34]. Clinical impairment, assessed at diagnosis through the ALSFRS-R score and FVC, is a predictive factor for the survival of patients with ALS [36].

One might expect that hospitalizations would be associated with inadequate home care, limited social support, and greater patient frailty, factors that could impact ALS patients’ survival [36]. Contrary to expectations, the number and duration of hospitalizations do not influence the survival of patients in our cohort. On the contrary, elderly individuals who chose not to undergo supportive procedures generally had shorter and less frequent hospitalizations. This is likely due to effective shared care planning, possibly facilitated by the implementation of the palliative care network [26,37], which is operational and widely accessible in our region. In fact, patients with a higher frequency of hospitalizations were more likely to take riluzole, suggesting that individuals who avoid supportive procedures and hospital admissions also tend to use fewer drugs. It can be ruled out that the increased risk of hospitalization is related to riluzole, a well-known drug with only minimal adverse effects [38].

In conclusion, hospitalizations are quite frequent among ALS patients, especially among younger ones who opt for respiratory and nutritional support. Given the frequency of hospitalizations, they present psychological and organizational challenges for patients and their families, as well as a significant economic burden on public health systems. Efforts should be made to minimize these hospitalizations as much as possible.

Notably, network care has been linked to fewer hospital admissions, less functional deterioration, and subsequently lower mortality in ALS. The use of hospital care for neurodegenerative and motor neuron diseases has declined in recent years, a trend that was accentuated by the SARS-CoV-2 pandemic [39].

As the main cause of hospitalization in ALS is disease progression and the need for life-support interventions, strengthening the home care network could positively impact the clinical trajectory of the disease, leading to a reduction in hospitalizations [40].

This study presents both strengths and limitations. Spanning a decade, it provides a long-term view of ALS patient hospitalizations, a dataset that is invaluable for observing trends and changes over time. Data from a population-based registry, integrated with multiple sources, ensures the completeness of the case study. The research delves into various aspects of hospitalizations, including reasons for hospital stays, such as gastrostomy and ventilation needs, length of stay, and frequency of hospitalizations. This detailed analysis facilitates a nuanced understanding of the healthcare needs of ALS patients and can inform tailored healthcare strategies.

However, the study’s retrospective design inherently limits it, because it is more susceptible to potential confounders. Additionally, we did not record data on further treatments received by patients during hospital admissions, which could impact the management and duration of hospitalization, nor did we collect information on patients’ quality of life. Finally, focusing exclusively on patients from Modena may restrict the generalizability of the findings to other countries with different healthcare systems or demographic characteristics.

## 5. Conclusions

This study addresses a relatively underexplored topic in the literature, providing an opportunity to examine the epidemiological, clinical, and survival implications of hospitalizations. Contrary to expectations, hospitalizations do not appear to be associated with shorter survival. This suggests that the decision to undergo hospitalization may be more influenced by social and familial factors than by the severity of the disease.

If the creation of specialized, multidisciplinary ALS centers and the enhancement of the home care network have demonstrated positive outcomes, improving both the quality of life and overall survival of patients [41], a further step forward would be to support patients’ families and further strengthen the home care network. This could help effectively manage complications at home, consequently reducing the need for hospital admissions or shortening the duration of hospital stays.

## Figures and Tables

**Table 1:** Clinical and demographic features of the patients included in the study according to sex.

Demographic and Clinical Features	Total, n (%), Mean [SD]	Males, n (%), Mean [SD]	Females, n (%), Mean [SD]	*p* Value
Education, y *	8.04 [4.49]	8.49 [4.87]	7.44 [3.88]	0.072
BMI at diagnosis, kg/m^2^ **	24.31 [ 4.31]	24.96 [3.67]	23.46 [4.91]	0.007
Age at onset, y ***	67.98 [12.09]	66.28 [11.68]	70.20 [12.32]	0.012
Age at diagnosis, y	69.02 [12.01]	67.38 [11.49]	71.17 [12.38]	0.013
Diagnostic delay, m ***	12.07 [11.02]	11.73 [12.03]	12.52 [9.58]	0.580
Presence of FTD	40 (16.06)	20 (14.18)	20 (18.51)	0.356
Site of onset:				0.311
Bulbar	95 (38.1)	46 (32.62)	49 (45.37)	
Upper limb	64 (25.7)	40 (28.36)	24 (22.22)	
Lower limb	76 (30.52)	46 (32.62)	30 (27.77)	
Respiratory	10 (4.01)	7 (4.96)	3 (2.77)	
Not known	4 (1.67)	2 (1.44)	2 (1.87)	
Phenotype:				0.217
Bulbar	94 (37.75)	46 (32.6)	48 (44.4)	
Classic	79 (31.72)	48 (34.04)	31 (28.70)	
Flail arm	15 (6.02)	6 (4.25)	9 (8.33)	
Flail leg	29 (11.64)	19 (13.47)	10 (9.25)	
UMNp	15 (6.02)	10 (7.09)	5 (4.62)	
Respiratory	13 (5.22)	10 (7.09)	3 (2.77)	
Not known	4 (1.63)	2 (1.46)	2 (1.93)	
ALSFRS-R total score at diagnosis, points $	38.99 [7.23]	40.26 [8.98]	37.31 [10.76]	**0.002**
Progression rate at diagnosis, points/month	1,21 [1.45]	1.21 [1.56]	1.22 [1.27]	0.959
FVC at diagnosis, % $$	82.91 [26.38]	83,43 [36.62]	82.18 [43.66]	0.741
PEG	122 (49.0)	64 (45.4)	58 (53.7)	0.193
Time from onset to PEG	28.14 [22.29]	30.30 [25.69]	25.81 [17.83]	0.270
NIV	133 (53.41)	80 (54.7)	53 (49.1)	0.230
Time from onset to NIV	27.75 [21.04]	29.03 [23.5]	25.83 [16.58]	0.391
IV	85 (34.14)	53 (37.6)	32 (29.6)	0.189
Time from onset to IV	30.78 [22.00]	31.43 [23.14]	29.67 [20.21]	0.726
Riluzole	224 (89.95)	132 (93.6)	92 (85.1)	**0.028**
Death	232 (93.17)	129 (91.4)	103 (95.37)	0.229
Survival from onset to death/tracheostomy/last observation, m	39.15 [37.66]	42.11 [40.04]	35.22 [34.05]	0.158
Total	249 (100)	141 (56.63)	108 (43.37)	

BMI = body mass index, FTD = frontotemporal dementia, UMNp = upper motor neuron-predominant, ALSFRS-R = ALS Functional Rating Scale—Revised, FVC = forced vital capacity, PEG = percutaneous endoscopic gastrostomy, NIV = non-invasive ventilation, IV = invasive ventilation. SD = standard deviation * Information available for 243/249 patients, ** information available for 238/249 subjects, *** information available for 245/249 subjects, $ information available for 241/249 subjects, $$ information available for 204/249 subjects.

**Table 2:** Clinical and demographic features of ALS patients according to number of hospitalizations.

Demographic and Clinical Features	Patients Never Hospitalized, n (%), Mean [SD]	Patients Hospitalized at Least Once, n (%), Mean [SD]	*p* Value ^£^	Patients Hospitalized at Least Twice, n (%), Mean [SD]	*p* Value ^$^
Sex, males	49 (34.75)	92 (65.25)	0.412	52 (36.17)	0.872
Education, y *	8.13 [4.32]	7.98 [4.59]	0.801	8.12 [4.74]	0.827
BMI at diagnosis, kg/m^2^ **	23.53 [4.26]	24.72 [4.29]	0.042	25.16 [4.23]	**0.021**
Age at onset, y ***	69.18 [12.12]	67.31 [12.07]	0.798	63.58 [11.61]	**<0.0001**
Age at diagnosis, y	70.31 [12.05]	68.27 [11.96]	0.197	64.56 11.40]	**<0.0001**
Diagnostic delay, m ***	12.99 [13.17]	11.55 [9.62]	0.330	11.73 [9.72]	0.722
Comorbidities:			0.359		0.663
3 or more	52 (56.52)	98 (62.42)		52 (58.43)	
Fewer than 3	40 (43.48)	59 (37.58)		37 (41.57)	
Site of onset:			0.076		0.347
Bulbar	32 (34.78)	63 (40.12)		39 (43.82)	
Upper limb	27 (29.34)	37 (23.56)		23 (25.84)	
Lower limb	26 (28.26)	50 (31.84)		23 (25.84)	
Respiratory	3 (3.26)	7 (4.48)		4 (4.50)	
Not known	4 (4.36)	0 (0)		0 (0)	
Fenotype:			0.142		0.311
Bulbar	32 (34.78)	62 (39.49)		38 (42.69)	
Classic	29 (31.52)	50 (31.84)		30 (33.70)	
Flail arm	6 (6.52)	9 (5.73)		6 (6.74)	
Flail leg	9 (9.78)	20 (12.73)		9 (10.11)	
UMNp	8 (8.69)	7 (4.45)		2 (2.24)	
Respiratory	4 (4.34)	9 (5.76)		4 (4.52)	
Not known	4 (4.37)	0 (0)		0 (0)	
PEG	20 (21.74)	102 (64.97)	<0.001	76 (85.39)	**<0.0001**
NIV	29 (31.52)	104 (66.24)	<0.001	65 (73.03)	**<0.0001**
IV	12 (13.04)	73 (46.50)	<0.001	60 (67.42)	**<0.0001**
Riluzole	76 (82.61)	148 (94.27)	0.003	87 (97.75)	**0.002**
Presence of FTD	14 (15.21)	26 (16.56)	0.781	13 (14.61)	0.908
Death	83 (90.22)	149 (94.90)	0.157	86 (96.63)	0.107
Survival from onset to death/tracheostomy/last observation, m	43.52 [46.04]	36.68 [31.89]	0.173	41.31 [36.68]	0.502
Total	92 (36.95)	157 (63.05)		89 (35.74)	

BMI = body mass index, FTD = frontotemporal dementia, UMNp = upper motor neuron-predominant, ALSFRS-R = ALS Functional Rating Scale—Revised, FVC = forced vital capacity, PEG = percutaneous endoscopic gastrostomy, NIV = non-invasive ventilation, IV = invasive ventilation. SD = standard deviation * Information available for 243/249 patients, ** information available for 238/249 subjects, *** information available for 245/249 subjects. ^£^: Patients never hospitalized versus patients hospitalized at least once ^$^: Patients never hospitalized versus patients hospitalized at least twice.

**Table 3:** Duration of hospitalizations in relation to the clinical–demographic characteristics of the patients.

Variable	Number of Patients	Length of Hospitalization, Day Mean [SD]	*p* Value
Sex			0.304
Females	65	29.47 [36.78]	
Males	92	35.12 [47.05]	
Age at onset, y:			**0.002**
65 y or less	59	44.23 [51.71]	
More than 65 y	98	26.46 [36.02]	
Riluzole:			0.063
Yes	148	34.36 [43.72]	
No	9	17.56 [31.72]	
PEG:			**<0.001**
Yes	102	51.53 [42.64]	
No	55	14.55 [34.68]	
NIV:			**<0.001**
Yes	104	46.44 [48.12]	
No	53	16.87 [29.01]	
IV:			**<0.001**
Yes	73	71.92 [47.94]	
No	84	12.32 [19.93]	
Emergency NIV:			**0.012**
Yes	33	62.84 [48.91]	
No	71	39.88 [46.46]	
Emergency IV:			0.277
Yes	68	73.35 [48.97]	
No	5	49.20 [14.53]	
Comorbidities:			0.965
Fewer than 3	99	32.52 [46.74]	
3 or more	150	32.76 [40.34]	

PEG = endoscopic percutaneous gastrostomy, NIV = non-invasive ventilation, IV = invasive ventilation.

**Table 4:** Univariate Cox regression analysis of tracheostomy-free survival in ALS patients of the study.

Variable	HR	95% CI	*p* Value
Sex (female/male)	0.81	[0.62–1.06]	0.12
Age at onset, y	1.03	[1.02–1.04]	**<0.001**
Diagnostic delay, m	0.94	[0.93–0.96]	**<0.001**
Onset			
Bulbar	1	reference	
Upper limb	10.7	[0.50–0.97]	**0.032**
Lower limb	0.59	[0.42–0.81]	**0.001**
Respiratory	1.16	[0.60–2.23]	0.662
Phenotype			
Bulbar	1	reference	
Classic	0.75	[0.55–1.02]	0.068
Flail arm	0.60	[0.34–1.05]	0.078
Flail leg	0.49	[0.31–0.78]	**0.002**
UMNp	0.25	[0.13–0.49]	**<0.001**
Respiratory	1.23	[0.68–2.20]	0.489
Presence of FTD	1.44	[1.01–2.04]	**0.041**
Weight loss at diagnosis, Kg	1.04	[1.02–1.07]	**<0.001**
ALSFRS-R score at diagnosis, points	0.96	[0.94–0.98]	**<0.001**
FVC at diagnosis, %	0.99	[0.98–1.00]	**0.002**
Riluzole treatment	0.71	[0.44–1.13]	0.147
At least 1 hospitalization	1.13	[0.86–1.49]	0.364
At least 2 hospitalizations	0.91	[0.70–1.20]	0.511
At least 3 hospitalizations	0.86	[0.65–1.21]	0.445
At least 4 hospitalizations	0.83	[0.58–1.19]	0.317
At least 5 hospitalizations	0.83	[0.54–1.29]	0.411
Total days of hospitalization	1	[0.99–1.00]	0.339
Number of admissions	0.98	[0.95–1.02]	0.414
Number of comorbidities	1.03	[0.97–1.11]	0.286

UMN-p = upper motor neuron-predominant; FTD = frontotemporal dementia.; ALSFRS-R = ALS Functional Rating Scale—Revised; FVC = forced vital capacity; HR = hazard ratio; CI = confidence interval. Independent prognostic factors for the length of tracheostomy-free survival in the multivariate analysis were age at onset (years) (HR = 1.02, 95% CI: 1.01–1.04, *p* < 0.001), phenotype (HR = 0.86, 95% CI: 0.77–0.96, *p* = 0.008), ALSFRS-R score at diagnosis (per 1 point) (HR = 0.97, 95% CI: 0.95–1.00, *p* = 0.052), and FVC at diagnosis (%) (HR = 0.99, 95% CI: 0.99–1.00, *p* = 0.019).

## Data Availability

The data are available from the authors upon reasonable request.
